# Metagenomic Antimicrobial Susceptibility Testing from Simulated Native Patient Samples

**DOI:** 10.3390/antibiotics12020366

**Published:** 2023-02-09

**Authors:** Lukas Lüftinger, Peter Májek, Thomas Rattei, Stephan Beisken

**Affiliations:** 1 Centre for Microbiology and Environmental Systems Science, University of Vienna, 1030 Vienna, Austria; 2 Ares Genetics GmbH, 1030 Vienna, Austria; 3 Doctoral School in Microbiology and Environmental Science, University of Vienna, 1030 Vienna, Austria

**Keywords:** antimicrobial resistance, antimicrobial susceptibility testing, machine learning, bioinformatics, NGS, clinical metagenomics

## Abstract

Genomic antimicrobial susceptibility testing (AST) has been shown to be accurate for many pathogens and antimicrobials. However, these methods have not been systematically evaluated for clinical metagenomic data. We investigate the performance of in-silico AST from clinical metagenomes (MG-AST). Using isolate sequencing data from a multi-center study on antimicrobial resistance (AMR) as well as shotgun-sequenced septic urine samples, we simulate over 2000 complicated urinary tract infection (cUTI) metagenomes with known resistance phenotype to 5 antimicrobials. Applying rule-based and machine learning-based genomic AST classifiers, we explore the impact of sequencing depth and technology, metagenome complexity, and bioinformatics processing approaches on AST accuracy. By using an optimized metagenomics assembly and binning workflow, MG-AST achieved balanced accuracy within 5.1% of isolate-derived genomic AST. For poly-microbial infections, taxonomic sample complexity and relatedness of taxa in the sample is a key factor influencing metagenomic binning and downstream MG-AST accuracy. We show that the reassignment of putative plasmid contigs by their predicted host range and investigation of whole resistome capabilities improved MG-AST performance on poly-microbial samples. We further demonstrate that machine learning-based methods enable MG-AST with superior accuracy compared to rule-based approaches on simulated native patient samples.

## 1. Introduction

Antimicrobial resistance (AMR) is a growing public health concern. The number of deaths due to bacterial infections is projected to exceed 10 million per year by 2050 [1]. Antimicrobial susceptibility testing (AST) is routinely performed using growth-based phenotypic methods, which require the culturability of the pathogen in question. With the availability of bacterial sequencing, methods that infer AMR based on whole-genome sequencing data have been developed and demonstrated to be accurate for many pathogens and antimicrobials [2].

Genomic AST software predicts AMR either through rule-based or machine learning (ML) algorithms. For the former, decision rules based on the presence of AMR markers in a pathogen’s genome are used to infer resistance [3,4]. For the latter, computational models are trained on a collection of pathogen genomes paired with reference AST data to learn the relationship between genomic information and susceptibility/resistance [5,6,7,8]. The performance of genomic AST methods has repeatedly been shown to match clinical diagnostic performance criteria for many organisms and antibiotics [3,4,5,9,10]. Critical for genomic AST is the taxonomic identification of a (poly-)microbial sample to select the appropriate set of decision rules or ML models. A further constraint is a requirement for the availability of whole genomes for prediction, for example, via pathogen isolation and whole-genome sequencing, incurring significant labor and limiting the time to result. Genomic AST on metagenomics data, i.e., directly from native specimens, offers shorter turnaround times but remains an unsolved challenge. Compared to isolate sequencing, metagenomics data encompasses sequencing reads from the host, the host’s normal flora, contaminant species, and one or more target pathogens of varying sequencing coverage and requires additional computational pre-processing.

Metagenomic AST (MG-AST) software could be based on decision rules broadly applicable to a range of pathogen taxa and combinations thereof or on ML classifiers trained on metagenomic sequencing data from diverse taxa to enable discrimination of the metagenomic context and accurate resistance calling. Both approaches are limited by the lack of clinical metagenomic data paired with reference AST information of constituent pathogens.

As a workaround, genomic AST software developed for isolate sequencing data could be enabled for MG-AST through appropriate pre-processing of sequencing reads, i.e., a classical genome-centric metagenomics workflow. Genome-centric metagenomics encompasses first the assembly of all reads from a metagenomic sequencing run into a singular large metagenomic assembly. Subsequently, contigs are split into metagenomic bins putatively representing genomes of distinct microbial populations in the input sample. This is performed using cues evident within assembled contigs pointing to their provenance, including but not limited to the following: tetranucleotide frequency, presence of universal marker genes, and differential coverage of contigs in the input read data [11]. Metagenomic assembly and binning establishes the mapping between genomic data and originating bacterial populations required by genomic AST software. One limitation is the accuracy and comprehensiveness of metagenomics bins. Studies have shown that the metagenomic assembly of plasmid contigs and AMR marker genes is significantly less effective than for general bacterial chromosomes [12], potentially leading to a degradation in the sensitivity of resistance predictions.

In this work, we investigate the performance of metagenomic binning for the application of MG-AST techniques on simulated clinical metagenomics data. While datasets exist with paired AST and WGS data of bacterial isolates [13], similarly characterized clinical metagenomic datasets are scarce and may not be optimal for MG-AST validation purposes as unculturable pathogens represent a significant fraction of clinical cases [14]. The set of organisms, which can be isolated from a native sample, may misrepresent true sample diversity and lead to faulty reference AST assignments to the whole metagenome. We instead opt for a simulation-based experimental setup where fully determined metagenomes are created to replicate the bacterial and host background of complicated urinary tract infections, containing reads derived from isolate genome assemblies with known AST status. This allows us to vary parameters pertaining both to sequencing itself, such as sequencing technology and sequencing depth, as well as downstream bioinformatic analysis, such as settings for metagenomic binning.

## 2. Results

### 2.1. Mono-Infection Scenarios

#### 2.1.1. Impact of Sequencing Depth and Human Background Reads on MG-AST Accuracy

For each *E. coli* genome from Ferreira et al. [10], five sets of 300 Mbp paired-end short read datasets were simulated with CAMISIM, containing an increasing fraction of on-target *E. coli* reads from 12.5% (7.5× coverage of the pathogen) to 96% (55× coverage of the pathogen). MG-AST was performed directly on metagenome-assembled genomes after the depletion of human reads by alignment to the human reference genome. Resistance prediction was performed using both the rule-based ResFinder 4 [3] and ML-based WGS-AST [5] methods (see Methods Section 4.6). No significant performance differences between MG-AST methods applied to metagenome assemblies and the originating isolate genome assemblies were apparent, and a drop in predictive accuracy was found only at the lowest assayed on-target coverage (7.5×) (Supplementary Table S1). This indicates that given a pathogen genome coverage of at least 15×, in silico depletion of reads aligning to the human reference genome, as well as the presence of any human-originating reads not removed by depletion, does not significantly affect the successful assembly of genomic features used by the tested methods.

#### 2.1.2. MG-AST Accuracy on Simulated *E. coli* cUTI Metagenomes

**Figure 1:**
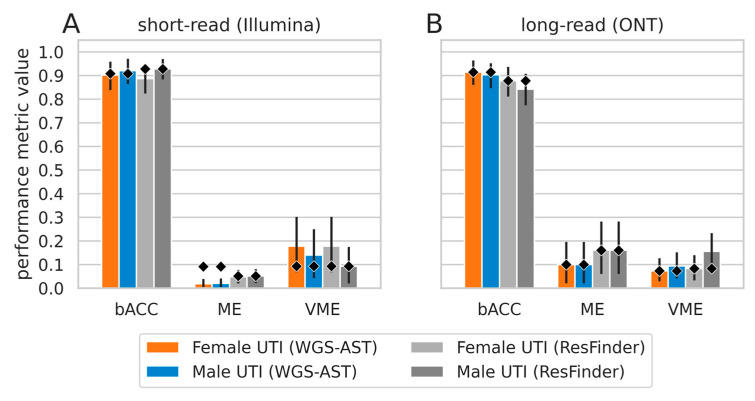
Performance metrics of MG-AST methods on simulated *E. coli* mono-infection complicated urinary tract infection (cUTI) metagenomes. The height of bars indicates the value taken on by balanced accuracy (bACC), major error (ME) and very major error (VME) in each investigated experimental setting. Metrics are aggregated over 5 antibiotic compounds. (**A**), performance on simulated short-read datasets. (**B**), performance on simulated Oxford Nanopore Technologies (ONT) sequencing datasets. Diamonds indicate baseline value taken on by the metric, obtained on isolate genome assemblies used to construct metagenomes. Error bars are 95% confidence intervals obtained by 1000× bootstrapping.

Using taxonomic profiles constructed from sequencing reads of two clinical septic urine samples derived from a female and from a male patient, we defined metagenome backgrounds to which *E. coli* reads from isolates with known AST status were inserted. For short-read data simulation, *E. coli* genome assemblies from [10] were used as the target pathogen, and the same size of the data apparent in the original sequencing run of the septic urine sample (1.18 Gbp) was simulated. For long-read metagenomes, the contiguity of assemblies from [10] was insufficient to simulate realistic long-reads from Oxford Nanopore Technologies (ONT). Instead, *E. coli* genomes assembled from long-read sequencing runs published in either PATRIC or NDARO databases were used [15,16]. Simulated metagenomes were preprocessed and assembled. From each simulated metagenome, a single high-quality metagenomic bin identified by Kraken2 as *E. coli* was extracted, and MG-AST methods were applied for each of the five selected target antibiotics using optimized MG-AST workflows (see Section 2.2.1 and Methods Section 4.4, Section 4.5 and Section 4.6). Classification performance metrics, namely, balanced accuracy (bACC) as well as a major error (ME) and very major error (VME) were computed against phenotypic AST results from originating bacterial isolates (see Methods Section 4.6). The aggregate clinical performance metrics of MG-AST showed no significant difference in bACC between baseline predictions on isolate genome assemblies and predictions on metagenomic bins (Figure 1). This indicates that, given sufficient sequencing depth, MG-AST can achieve performance comparable to isolate-sequencing-based genomic AST. This was the case both for metagenomes modeled after a complicated UTI of a male patient with a high relative fraction of pathogen reads and low bacterial and human background (<1% and 15%, respectively), as well as for metagenomes derived from septic urine of a female patient, containing a high percentage (88%) of reads mapping to either human background or vaginal normal flora organisms.

### 2.2. Co-Infection Scenarios

#### 2.2.1. MG-AST for Co-Infections with Metagenomic Binning

**Figure 2:**
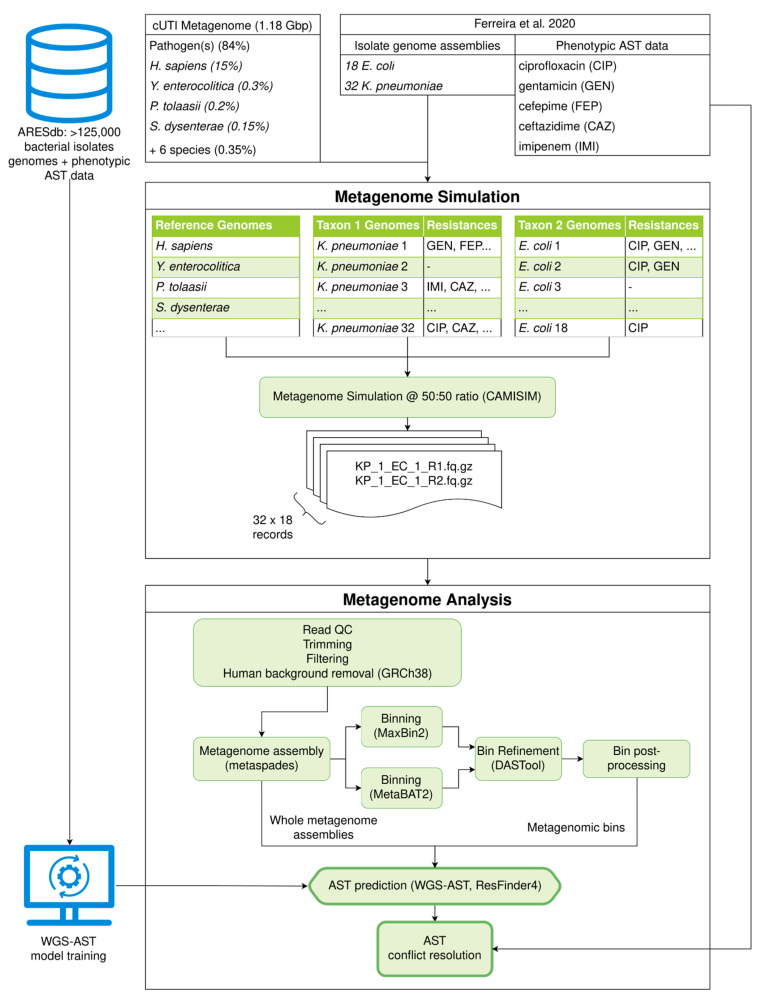
Experimental layout of *E. coli/K. pneumoniae* co-infection MG-AST experiment, encompassing data selection, metagenome simulation, binning, and MG-AST workflow. Genome assemblies and AST data from Ferreira et al., 2020 [10].

We investigated whether metagenomic binning-based workflows can support MG-AST for the resolution of two-species cUTI co-infections. We first investigated the co-infection of the two most common cUTI pathogens, *E. coli* and *Klebsiella pneumoniae*, at equimolar concentrations (Figure 2). The genome assembly and binning workflow were applied to 576 simulated cUTI coinfections derived from 18 *E. coli*, and 32 *K. pneumoniae* isolate genome assemblies. 

**Figure 3:**
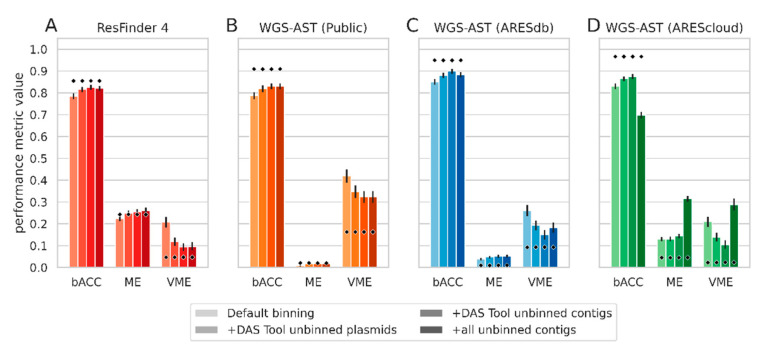
Performance metrics of genomic AST methods on metagenomic bins derived from simulated *E. coli/K. pneumoniae* co-infection cUTI metagenomes using different binning and AST prediction settings. Height of the bars indicates values taken on by bACC, ME, and VME in each investigated experimental setting. Metrics are averaged over 5 antibiotic compounds. Legend elements correspond with bars in each subfigure by color saturation, from lightest to darkest color. Diamonds indicate the mean value taken on by metrics of the tested method on isolate genome assemblies used to construct the metagenomes. (**A**), ResFinder 4 performance; (**B**), Performance of WGS-AST models from [5] trained on public data; (**C**), Performance of WGS-AST models trained analogously to [5] but including proprietary data from ARESdb [13]; (**D**), Performance of default AREScloud [17,18] WGS-AST models (optimized for application to isolate genome assemblies) trained on data as in C but using an extended feature space. bACC, balanced accuracy. ME, major error. VME, very major error. Error bars are 95% confidence interval obtained by 1000× bootstrapping.

MG-AST prediction was performed using ResFinder 4 as well as previously published WGS-AST model artifacts for *E. coli* and *K. pneumoniae,* which were trained on publicly available datasets (termed “WGS-AST (Public)”) [3,5]. Results indicated that relative to predictions on isolate genomes, a significant increase in very major error (VME) by 15–20 percentage points (pp) was apparent with the default binning strategy. This increase was seen for both rule-based ResFinder 4 and ML-based WGS-AST predictions (Figure 3A,B, “Default binning”). We hypothesized that this uniform loss of sensitivity was due to a failure to include AMR marker genes in metagenomic bins. AMR genes commonly reside on mobile genetic elements (MGEs), which have been shown to be binned with poor efficiency owing to differential nucleotide composition and copy number compared to the bacterial chromosome [12]. To test this hypothesis, we performed an alignment-based search of the set of AMR marker genes identified in whole metagenome assemblies and the metagenomic bins versus the isolate genomes used in metagenome simulation. On average, isolate genomes contained 212 AMR markers, and, on average, 10.2% of AMR markers identified in an isolate genome assembly were absent in the corresponding metagenomic bin. Conversely, only 0.36% of expected AMR markers were absent from whole metagenome assemblies. This suggests that while metagenome assembly was successful in capturing the resistome capabilities, AMR markers were lost or misassigned during metagenomic binning. 

To counter this and improve the predictive accuracy of MG-AST, we devised post-processing schemes for metagenomic bins. We enriched bins with high-quality contigs excluded at the binning refinement stage by DAS Tool, optionally considering only contigs matching to known plasmids (Figure 3, “+DAS Tool unbinned contigs” and “+DAS Tool unbinned plasmids”, respectively). This reduced VME significantly, on average, by 10 pp while increasing major error (ME) by 2 pp. Retraining ML classifiers on a larger and more diverse dataset of bacterial isolate sequencing data derived from ARESdb (models termed “WGS-AST (ARESdb)” [13] yielded a significant drop in VME on metagenomic bins by nearly 20 percentage points down to 15% (Figure 3C). We then applied WGS-AST classifiers operating on an extended feature space, which utilizes DNA k-mer counts instead of DNA k-mer occurrences, as well as protein k-mer counts and protein mutational scoring features (Figure 3D, models termed “WGS-AST (AREScloud)”) [17,18]. While performing well on isolate genomes themselves, overall performance on metagenomic bins was below that of “WGS-AST (ARESdb)” models. Complex feature space models proved unstable upon the addition of low-quality unbinned contigs not included in any initial metagenomic bins, causing a degradation of bACC by nearly 20 pp (Figure 3D, “+all unbinned contigs”).

We thus chose models trained as in [5] but with extended training data for experimentation with metagenomic data, maintaining prediction with extended feature space models on isolate genome assemblies as our baseline performance. We chose to continue with postprocessing metagenomic bins by the addition of all contigs excluded by the DAS Tool, a strategy that exhibited overall optimal predictive performance across binning and MG-AST algorithm selection (Figure 3C, “+DAS Tool unbinned contigs”). Using this binning post-correction strategy, the average fraction of expected but missing AMR markers per metagenomic bin was lowered from 10.2% to 8.3%.

**Figure 4:**
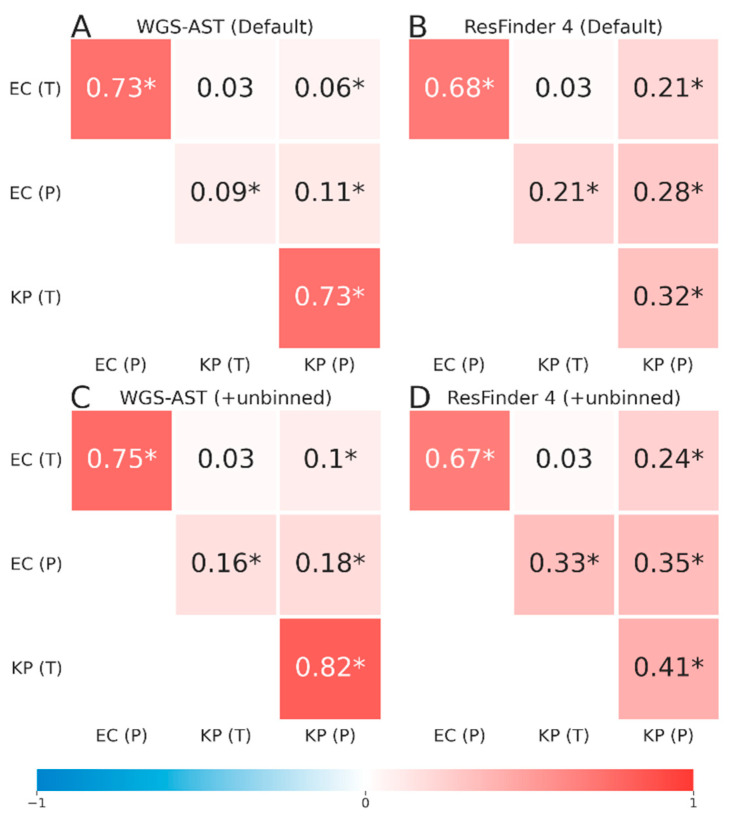
Pearson correlation of True and Predicted AST status of metagenomic bins in *E. coli*/*K. pneumoniae* co-infection metagenomes. (**A**,**B**), correlation for WGS-AST and ResFinder 4 classifiers, respectively, on metagenomes binned with default settings. (**C**,**D**), correlation for WGS-AST and ResFinder 4 classifiers, respectively, on metagenomic bins with added DAS Tool unbinned contigs. EC, *Escherichia coli*. KP, *Klebsiella pneumoniae*. (T), true AST status. (P), predicted AST status. Correlations with significance at *p* < 0.05 indicated with asterisk.

While overall predictive performance benefited from the enrichment of metagenomic bins with high-quality unbinned contigs, it may introduce spurious correlations between predictions of individual bins in a metagenome. To quantify the magnitude of this effect, we calculated the Pearson correlation of predicted and true resistance status of bins within metagenomes with default binning and binning under the inclusion of unbinned contigs. Results showed a significant positive correlation across the two major pathogen bins within metagenomes already for the default binning strategy (Figure 4A,B), indicating the transfer of resistance markers. The strength of spurious correlations was increased with the chosen binning optimization strategy (Figure 4C,D), which was deemed acceptable due to the overall increase in predictive accuracy and correlation strength between the true and predicted phenotypes of individual bins. Overall, ResFinder 4 predictions exhibited stronger spurious correlation across bins than WGS-AST predictions.

Using thus optimized settings, WGS-AST and ResFinder 4 reached relative balanced accuracy (bACC) within 5 and 3 percentage points of predictive performance on isolate genome assemblies, respectively. The absolute bACC of WGS-AST exceeded that of ResFinder 4 by 9.4 and 7.5 pp on isolate genome assemblies and metagenomic bins, respectively. This was unsurprising, as no in silico AST panel existed in ResFinder 4 for *K. pneumoniae,* and instead low-confidence taxon-unspecific ResFinder calls were used. Performance differences between isolate and metagenomic bins for rule- and ML-based methods in *E. coli* were non-significant (see Supplementary Table S2).

Several metagenomic binning tools used in this work utilize taxon-specific genomic signatures such as tetranucleotide frequencies [19] and single-copy marker genes [20] to accurately split contigs into draft metagenomic bins. We thus investigated the effect of taxonomic relatedness of pathogens on the effectiveness of binning-enabled MG-AST. We repeated co-infection metagenome experiments with two more distantly related taxa, namely, *A. baumannii* and *K. pneumoniae*, using optimized binning settings derived from the previous experiment. In total, 608 metagenomes were simulated using 19 *A. baumannii* and 32 *K. pneumoniae* isolate genomes. As there was no in silico AST panel for either organism in ResFinder 4, the resulting predictive accuracy of ResFinder 4 in this experiment was significantly below that of WGS-AST on both isolate assemblies and metagenomic bins. Thus, focusing on WGS-AST performance, we observed bACC to be 3.4 pp below that of the corresponding isolate genome WGS-AST. This constitutes a 1.5 pp increase compared to the previous experiment, which investigated two more closely related taxa. Importantly, this increase was not solely due to higher overall performance of WGS-AST on *A. baumannii* compared to *E. coli*, as the performance on *K. pneumoniae* metagenomic bins was improved by 3.4 pp over the previous experiment (see Supplementary Table S2). We again compared the AMR marker sets apparent in metagenomic bins and whole metagenomes to those of their corresponding input isolate genome assemblies. On average, isolate genomes contained 172 AMR marker genes, and only on average 1.66% of markers were lost during processing of *A. baumannii*/*K. pneumoniae* metagenomic bins, with, on average, 0.17% of expected markers absent from whole metagenome assemblies. These results indicate that metagenomic binning was more successful in correctly assigning AMR markers than in the previous experiment.

Offerings by Illumina remain the dominant sequencing technology used in clinical applications, with 90% of sequencing runs newly submitted to SRA in 2021 having been performed on Illumina devices [21]. However, long-read sequencing paradigms spearheaded by Pacific Biosciences (PacBio) and ONT are increasingly evaluated as alternatives. We repeated co-infection experiments of *E. coli* and *K. pneumoniae* using simulated metagenomic long reads and found that the optimal binning strategy devised with short-read metagenomes caused a relative increase in false resistance calls. We reasoned that since genome contiguity achieved with long-read sequencing data was significantly higher than with short-read data, the severity of misassigning unbinned contigs would be increased as well. We thus devised an additional binning correction scheme. We applied the MOB-recon tool to all finished bins derived from long-read metagenomes, noting the predicted taxon of origin for thus identified plasmid contigs. We marked any contig exhibiting a discrepant taxon of origin compared to its containing metagenomic bin as re-assignable. Reassignment was then performed for contigs where a metagenomic bin matching the predicted taxon of origin (up to genus level) was identified in the metagenome too. Using this strategy, a relative bACC of AST determination from metagenomic bins compared to isolate genome assemblies of 4.6 and 6 percentage points was observed for WGS-AST and ResFinder 4, respectively (see Supplementary Table S2). This is comparable to performance differences observed for short-read data. Analysis of AMR marker sets obtained from metagenomic bins, resistomes and isolate assemblies showed that inclusion of marker genes into metagenomic bins was improved compared to short-read experiments. While the overall average number of AMR markers identified per isolate assembly was comparable with 215, on average, only 1.82% of marker genes were missing from metagenomic bins. Whole metagenome assemblies were missing a fraction of AMR markers (0.29%), comparable to short-read experiments.

Finally, we investigated three-species cUTI co-infection scenarios using ten genomes per pathogen taxon, which were selected manually from datasets used in short-read metagenome experiments (see Methods Section 4.2). Performance metrics indicated a relative performance differential of 5 and 13 percentage points in bACC compared to isolate genomes for WGS-AST and ResFinder 4, respectively (see Supplementary Table S2).

#### 2.2.2. Resolving Co-Infection Scenarios with Resistome Analysis

Genome-level AST assignments are desirable, particularly in the presence of commensal taxa, which may contribute to the overall resistome of the sample while not being of major diagnostic importance. Given sufficiently low metagenome complexity and sufficient coverage for retrieval of high-quality metagenomic bins, MG-AST performance approached accuracy levels achieved on isolate genome assemblies. As is apparent from the investigation of AMR marker presence, loss of genomic information at the binning stage plays a significant role in explaining residual performance differences. Thus, in addition to AST calls made at the metagenomic bin level, we evaluated the performance of genomic AST methods directly on whole metagenome assemblies. Since taxon assignments are required by the assayed genomic AST methods, we applied each method to each metagenome for each pathogen taxon identified by binning. To obtain a single resistance or susceptibility call per tested compound, we evaluated two schemes to merge predictions. Firstly, we evaluated merging predictions such that any resistance call led to overall resistance for the whole resistome (the “max” strategy). However, WGS-AST classifiers yield not only a binary S/R call but instead the calibrated probability of resistance (0.0–1.0), which can be interpreted as the confidence of the ML model in its prediction, given the supplied data. We thus evaluated another strategy termed “most certain”, where WGS-AST predictions were merged by selecting the prediction with the confidence score furthest from the decision boundary of 0.5. Resistome AST performance metrics showed the “most certain” strategy to be slightly superior to the “max” strategy with respect to overall bACC but exhibiting a higher overall VME. The “max” strategy exhibited a bACC drop relative to averaged isolate performance by 3.3 percentage points, slightly better than individual AST calls made on metagenomic bins (Table 1 and Supplementary Table S2).

## 3. Discussion

In this work, we investigated the potential of applying genomic AST methods to sequencing data derived from native clinical samples. We confirm that initial data quality, as well as the accuracy of metagenomic binning are of pivotal importance for the performance of downstream applications such as genomic AST. We derived a metagenomic binning workflow, which prioritizes the inclusion of AMR marker genes over bin purity to optimize the balanced accuracy of AST determination. We present a workflow that, for native samples with low bacterial complexity and sufficient on-target sequencing depth, exhibits on-par performance with genomic AST on isolate sequencing data. Investigating UTI co-infections, we find that using the same optimized workflow, an average performance differential of between 3.4 and 4.6 percentage points bACC was apparent, mostly caused by an increase in false susceptibility AST calls. 

Correct and complete binning of AMR marker genes and accuracy of AST determination were impaired by pathogen-relatedness in co-infection experiments. This limitation of default genome binning techniques could be partially addressed for the purpose of genomic AST by supplementing metagenomic bins with high-quality unbinned genomic material and by reassignment of putative plasmid contigs with the known taxon of origin. In this work, we experimented with two widely used metagenomic binning tools. Further improvements may be possible by adding additional binning signals to serve as input to DAS Tool. We additionally show that an improvement in the sensitivity of prediction can be realized by whole resistome analysis, albeit sacrificing the ability to distinguish the taxon from which resistance originated.

Comparing the performance of MG-AST on short-read and long-read metagenomic data, we find that relative MG-AST performance was comparable. We note the improved recovery of AMR markers into metagenomic bins with simulated long-read data, which may support further improvements in genomic AST accuracy in the future.

We compared two previously published genomic AST algorithms designed to operate on isolate sequencing data. Results showed that while performance in simple mono-infection settings of *E. coli* was comparable, our ML-based technique performed better on bins derived from two- and three-species co-infection metagenomes. ResFinder 4 exhibited an a priori lower performance on isolate genomes of *K. pneumoniae* and *A. baumannii*, taxa for which the tool provides no manually curated databases and instead falls back on taxon-unspecific prediction based on the presence/absence of resistance genes. However, the relative performance drop from *E. coli* isolate genomes to metagenomic bins was larger for ResFinder 4. We show that the spurious correlation between true and predicted AST statuses across genomes was more severe for ResFinder 4 predictions than for WGS-AST classifiers, confirming that incorrectly binned genomic information differentially affected the two methods. A potential explanation for this divergence is the reliance of ResFinder on a defined set of decision rules predicated on the identification of causal AMR markers identified within the genome, while WGS-AST classifiers utilize a larger number of decision criteria (DNA k-mer presence patterns learned from training data). In principle, this allows WGS-AST classifiers to use all AMR-contributing genomic information present in a sample and to recover from the loss of a single resistance-contributing gene.

In this work, we show that, under the tested conditions, MG-AST performance is comparable to genomic AST from bacterial isolate sequencing. Cultivation and isolation are typically the most time-consuming parts of a classical phenotypic AST workflow, requiring up to several days, depending on the growth rate of the pathogen(s) [22]. Metagenomic pathogen ID and AST promise to eliminate these steps entirely, thus removing a hard lower limit on the time-to-result of AST. At the same time, long-read sequencing devices allow the streaming of data to downstream analyses already during sequencing and without user intervention, potentially enabling overall processing from sample preparation to MG-AST and ID results in a single day [23,24]. The total cost of either classical phenotypic AST or MG-AST significantly depends on the applied technologies and workflows. However, the overall positive impact of microbial sequencing on healthcare economics has been shown in several studies [25,26].

## 4. Materials and Methods

### 4.1. Taxonomic Profiles, Genomic and Antimicrobial Susceptibility Data 

Relative abundances of contaminant species and human background read used in simulated cUTI metagenomes were modeled after bulk Illumina sequencing runs of a recurrent clinical septic urine mono-infection of *Escherichia coli* sampled from a male patient [27] and a septic urine mono-infection of *Enterococcus faecalis* sampled from a female patient (see Supplementary Table S3). Taxonomic profiles for metagenome simulation were constructed with Kraken2 [28]. Sequencing reads and the number of reads assigned at the species level were noted for all species thus identified. Species with less than 1000 reads were discarded. Finally, reads identified as belonging to the primary pathogen at the species level were removed from the profile. The fraction of remaining bacterial reads as well as of human background reads was computed as the number of reads assigned to that taxon divided by the total number of reads in the sequencing run.

Reference genomes of background and contaminant species were downloaded from NCBI RefSeq (see Supplementary Table S3) Genome assemblies of *E. coli*, *K. pneumoniae,* and *A. baumannii,* which were used in simulation of metagenomes, were retrieved from ARESdb (see Supplementary Table S4 for a list of corresponding NCBI accessions). All AST data as well as genome assemblies used for training of WGS-AST machine learning models were retrieved from ARESdb. Genome assemblies used for model training were selected to exclude genomes used in metagenome simulation [13].

### 4.2. Metagenome Simulation

Short-read simulation was performed using CAMISIM 1.3 [29] and the ART [30] backend with the default MBARC-26 error profile, causing the simulator to output 2 × 150 bp paired-end reads with an error profile modeled after the Illumina HiSeq 2500 sequencer. Long-read simulation was performed using PBSIM2 [31]. To obtain a set of metagenomic long reads required by PBSIM2 to sample error profiles and read lengths from, raw FAST5 files with ENA accession number ERR2887850 (an ultra-deep ONT sequencing run of a mock microbial community [32]) were downloaded and basecalled with guppy 6.2.7 using the arguments “-c dna_r9.4.1_450 bps_sup.cfg –compress_fastq -q 32000 --chunks_per_runner 768”. Long read simulation was then performed for each input genome of a to-be-created metagenome using the sampling-based simulation method and arguments “--difference-ratio 23:31:46 --depth X” where X was the desired coverage depth in the resulting metagenome.

Genome assemblies from Ferreira et al. [10] (for experiments with simulated short-read metagenomes) and from public AMR databases (for experiments with simulated long-read datasets) were used to simulate reads. The Ferreira et al. dataset in particular was selected as it provides uniformly sequenced and assembled genomes for three key uropathogenic species. For the investigation of two- and three-species cUTI co-infections, genome assemblies were deduplicated on the multi-locus sequence type (MLST), and genomes of the two or three species under investigation were paired up to form the cartesian product of the input genome sets. Metagenomic sequencing reads were then simulated using the male patient cUTI species profile such that the overall fraction of reads occupied by *E. coli* in the original sequencing run was shared among the pathogen genomes in a ratio determined by the experimental setup. For the simulation of three-species metagenomes, ten isolates were selected from isolates of each species previously used in two-species metagenomes. Using the five antibiotic compounds investigated in this work, we defined the following three categories of samples: “highly susceptible”, featuring ≤ 1 resistant calls; “highly resistant”, featuring ≥ 4 resistant calls; “mixed”, featuring 2 or 3 resistant calls. For each species, the proportions of samples falling into each of these categories were calculated, and samples were picked in a random stratified manner to best represent the original proportions in the retained ten samples, requiring at least one sample to be picked in each category.

### 4.3. WGS-AST Model Selection and Training

WGS-AST model artifacts trained on public data from Lüftinger et al. [5] (“WGS-AST (Public)”) using the XGBoost algorithm [33] were selected to cover five major classes of antibiotic compounds (selecting the compounds gentamicin, ciprofloxacin, cefepime, ceftazidime, and imipenem) and the three pathogens commonly encountered in cUTI, which were used in metagenome simulation. To investigate the impact of training set size on the ML model performance, models were retrained on ARESdb, a large and diverse set of bacterial isolates [13] as described in [5] (models being referred to as “WGS-AST (ARESdb)”). For retrieval of optimal WGS-AST predictions from short-read isolate genome assemblies and to investigate the impact of model feature space complexity on the accuracy of MG-AST predictions, we selected models with matching compound profiles currently deployed to the AREScloud web application [18] (models referred to as “WGS-AST (AREScloud)”). These models were trained with data from ARESdb equivalent to “WGS-AST (ARESdb)” models but using an extended feature space described in [17].

### 4.4. Pre-Processing and Assembly of Simulated Metagenomes

Simulated metagenomic reads for each experimental condition and each combination of input genome assemblies were pre-processed and assembled into whole metagenome assemblies using one of two workflows, depending on the sequencing technology. For simulated paired-end short read data, reads were first subjected to depletion of human background reads with Bowtie 2.4.5 [34], retaining only reads which did not align concordantly to the human GRCh38 reference genome. Subsequently, reads were quality filtered and trimmed using Trimmomatic 0.39 [35] with parameters “ILLUMINACLIP:adapters.fa:2:30:10 LEADING:10 TRAILING:10 SLIDINGWINDOW:4:15 MINLEN:36”. Host-depleted and filtered reads were then assembled using SPAdes 3.15.4 [36] with the metaspades.py workflow and default settings. For simulated single-end long-read data, FASTQ files were first subjected to depletion of human background reads with Minimap 2.24 [37], retaining only reads which did not align with the human GRCh38 reference genome. Subsequently, reads were quality filtered by nanofilt 2.8 [38], with parameters “-q 7”. Host depleted and filtered reads were then assembled using Flye 2.9 [39] with parameters “--iterations 2 --meta --nano-hq” and assemblies were polished with Oxford Nanopore Medaka 1.6.1 using parameters “-m r941_min_sup_g507” [32,40].

### 4.5. Metagenomic Binning and Taxon Assignment

We adopted the best-practices nf-core/mag computational pipeline for the purpose of metagenomic binning [41]. Assembled contigs were grouped into metagenomic bins using both MaxBin 2.2.7 [42] with default settings and MetaBAT 2.15 [19] with parameters “—saveCls –seed 42 –minContig 1500”. For metagenomic binning of assemblies derived from simulated long reads, use of contig coverage information was disabled by passing a zeroed-out coverage matrix to MaxBin and by not passing the coverage matrix to MetaBAT. All bins thus obtained were refined by DASTool 1.1.5 [20] with parameters “--score_threshold 0.3 --write_bins --write_unbinned --write_bin_evals”, yielding deduplicated high-quality metagenomic bins as well as unbinned contigs. Post-processing of metagenomic bins was performed according to the experimental setting. Since downstream MG-AST analyses require a taxonomy to be assigned to input samples, Kraken 2 [28] was then applied to metagenomic bins using a Minikraken database, and taxonomy was assigned by scoring the sum of nucleotides assigned at the species level across all sequences in the metagenomic bin and selecting the most highly abundant taxon. Taxonomic assignments were transferred to the originating whole metagenome assemblies (allowing for the potential assignment of multiple species to a single metagenome assembly). To obtain host taxon assignments of plasmids, the MOB-recon tool from MOBsuite 3.1.0 [43] was used with default settings.

### 4.6. MG-AST Analysis

Whole genome assemblies and derived metagenomic bins were grouped by their assigned taxon. WGS-AST predictions with “WGS-AST (Public)” and “WGS-AST (ARESdb)” models were applied as in [5]. WGS-AST models trained with an extended feature space encompassing protein k-mers, AMR marker detection, and protein mutation scoring, termed “WGS-AST (AREScloud)”, were applied as in [17]. ResFinder 4 [3] was applied to all input fasta files, with default settings and using both ResFinder and PointFinder databases as available. Resistance calls made by WGS-AST and ResFinder were scored against reference phenotypic AST to obtain absolute clinical performance metrics, namely, balanced accuracy (bACC, equivalent to the mean of sensitivity and specificity), major error (equivalent to 1-specificity) and very major error (equivalent to 1-sensitivity). Metrics were then also compared with optimal baseline performance metrics obtained on isolate genome assemblies used in metagenome simulation to gauge any performance penalty incurred by the experimental setting. Unless noted otherwise, WGS-AST predictions were obtained using “WGS-AST (ARESdb)” models with the exception of baseline WGS-AST predictions for short-read isolate genome assemblies, for which predictions were obtained using “WGS-AST (AREScloud)” models. AMR marker genes were determined in genome assemblies and metagenomic bins as previously described [44].

## 5. Conclusions

Using a simulation-based approach, we show that existing genomic AST methods can achieve near-equivalent performance on metagenomic and isolate sequencing data for several pathogens associated with cUTI infections. For patient samples exhibiting clinically relevant pathogen load for cUTIs and low sample complexity, MG-AST methods may reduce time-to-result and complement routine AST. Pending in vitro validation, we believe our results guide further research into the application of clinical metagenomic sequencing for infectious disease diagnostics.

## Figures and Tables

**Table 1:** Summary statistics of genomic AST methods applied to simulated short-read metagenomes derived from data published in [10]. Performance metrics for metagenomic bins and isolate assemblies are aggregated over all short-read experiments (male and female *E. coli* mono-infection, *K. pneumoniae/E. coli* co-infection, *K. pneumoniae/A. baumannii* co-infection, and three-species co-infection) and 5 assayed antibiotic compounds. Metrics for resistomes are aggregated over resistome predictions as above and were scored against the true resistome as defined by the AST status of genomes used in the metagenome’s simulation. Values in brackets indicate 95% CI obtained by 1000× bootstrapping.

Method	Data Type	bACC	ME	VME
WGS-AST (AREScloud)	isolate assembly	0.956 (0.941–0.969)	0.046 (0.032–0.061)	0.042 (0.021–0.071)
WGS-AST (ARESdb)	metagenomic bins	0.915 (0.912–0.919)	0.057 (0.054–0.061)	0.112 (0.106–0.119)
WGS-AST (ARESdb)	resistome (max)	0.923 (0.917–0.928)	0.136 (0.126–0.147)	0.019 (0.016–0.022)
WGS-AST (ARESdb)	resistome (most certain)	0.926 (0.922–0.931)	0.023 (0.019–0.029)	0.124 (0.116–0.131)
ResFinder 4	isolate assembly	0.811 (0.781–0.837)	0.184 (0.156–0.213)	0.195 (0.149–0.246)
ResFinder 4	metagenomic bin	0.711 (0.706–0.717)	0.274 (0.267–0.28)	0.303 (0.294–0.312)
ResFinder 4	resistome (max)	0.812 (0.804–0.819)	0.284 (0.269–0.298)	0.093 (0.086–0.1)

## Data Availability

Sequence data analyzed in this study is available at NCBI GenBank (see Supplementary Table S4 for a list of accessions). Simulated sequencing reads generated for this work are available upon request.

## Supplementary Materials

The following supporting information can be downloaded at: https://www.mdpi.com/article/10.3390/antibiotics12020366/s1, Supplementary Table S1: Impact of coverage and human background reads on genomic AST performance; Supplementary Table S2: Per-species performance metrics of genomic AST experiments performed in this work; Supplementary Table S3: Metagenome profiles used in construction of simulated metagenomes; Supplementary Table S4: NCBI GenBank accessions of isolate genomes used in metagenome simulation.
